# Real-World Use of Opicapone in Management of Patients with Parkinson’s Disease and Early Motor Fluctuations in Spain: 12-Month Interim Analysis of REONPARK Study

**DOI:** 10.3390/brainsci16090961

**Published:** 2026-09-11

**Authors:** Lydia López-Manzanares, María Cerdán Sánchez, Berta Solano Vila, Tania Delgado Ballestero, Rocío García-Ramos, Juan García Caldentey, Ana Rodríguez-Sanz, Jesús Olivares Romero, Itziar Gaston, Mar Carmona-Abellán, Marina Mata Álvarez-Santullano, Raquel Vázquez Picón, Marta Rodríguez-De Miguel, Iciar Tegel Ayuela, Diogo Magalhães, Joerg Holenz, on behalf of the REONPARK Study Group

**Affiliations:** 1 Department of Neurology, Hospital Universitario La Princesa, 28006 Madrid, Spain; 2 Department of Neurology, Hospital Universitario Santa Lucia, 30202 Cartagena, Spain; 3 Department of Neurology, Hospital Josep Trueta, 17007 Girona, Spain; 4 Department of Neurology, Hospital Santa Caterina, 17004 Girona, Spain; 5 Consorci Corporació Sanitària Parc Taulí, Hospital Universitari Parc Taulí, 08208 Sabadell, Spain; 6 Department of Neurology, Hospital Clínico San Carlos, 28040 Madrid, Spain; 7 Centro Neurológico Oms 42, 07003 Palma de Mallorca, Spain; 8 Department of Neurology, Hospital Universitario La Paz, 28046 Madrid, Spain; 9 Neurology Service, Hospital Universitario Torrecárdenas, 04009 Almería, Spain; 10 Department of Neurology, Complejo Hospitalario de Navarra, 31008 Pamplona, Spain; 11 Department of Neurology, Hospital Universitario Basurto, 48013 Bilbao, Spain; 12 Department of Neurology, Hospital Universitario Infanta Sofia, 28702 S. Sebastian de los Reyes, Spain; 13 Neurology Department, Hospital Universitario Galdakao-Usansolo, 48960 Bizkaia, Spain; 14 Laboratorios Bial, S.A., 28016 Madrid, Spain; 15 Bial—Portela CA, S.A., 4785 Coronado, Portugal

**Keywords:** COMT inhibitors, early fluctuations, OFF time, opicapone, Parkinson’s disease

## Abstract

**Highlights:**

**What are the main findings?**

- In patients with Parkinson’s disease and early motor fluctuations, opicapone initiation was associated with sustained improvements in motor symptoms and motor complications over 12 months, with clinically meaningful reductions in MDS-UPDRS Part III and IV scores.
- OFF time was reduced, while ON-time quality was maintained, with more than one-third of patients reporting no OFF periods and most patients remaining on stable levodopa doses after 12 months.

**What are the implications of the main findings?**

- These findings support the early use of opicapone after the onset of motor fluctuations as a strategy to achieve sustained motor control without the need for substantial levodopa dose escalation.
- Early recognition and treatment of wearing-off symptoms may help optimize long-term symptom management while preserving the quality of ON time in routine clinical practice.

**Abstract:**

Objective: To evaluate the sustained real-world effectiveness and safety of catechol-O-methyltransferase (COMT) inhibitors added to levodopa in patients with Parkinson’s disease (PD) presenting early motor fluctuations. Methods: REONPARK is an ongoing, multicenter, prospective observational study conducted in Spain. Adult patients with PD and end-of-dose motor fluctuations for <2 years initiating a COMT inhibitor were included. Outcomes assessed at 3, 6, and 12 months included Clinician and Patient Global Impression of Change (CGI-C, PGI-C), Movement Disorder Society–Unified Parkinson’s Disease Rating Scale (MDS-UPDRS), Wearing-off Questionnaire (WOQ-19), Non-Motor Symptoms Scale (NMSS), Parkinson’s Disease Questionnaire-8 (PDQ-8), and safety. This article presents the 3-, 6-, and 12-month planned interim analysis of the study. Results: A total of 143 patients were included (99.3% treated with opicapone), of whom 138, 131, and 89 patients completed the 3, 6, and 12 months of follow-up, respectively. Levodopa doses remained stable in most patients (73% at 12 months). Significant and sustained improvements were observed in MDS-UPDRS Parts III and IV scores, as well as the total MDS-UPDRS score over 12 months, with greater likelihood of Part IV improvement in patients receiving <4.5 mg/kg/day of levodopa. OFF time decreased by 1.6–2.0 h from baseline. ON-time quality was maintained over time with 97.7% of patients reporting no or minimal functional impact from dyskinesia, while 47.7% of patients had no impact from motor fluctuations and 36.4% of patients no longer experienced OFF periods at 12 months. PDQ-8 scores remained stable and no overall benefit on total non-motor symptoms burden was demonstrated, despite improvements in selected domains among patients with a higher baseline non-motor symptoms burden. WOQ-19 scores improved significantly. Conclusions: In PD patients with early motor fluctuations, opicapone initiation was associated with sustained improvements in motor outcomes and reduced OFF time, and maintained the ON-time quality over 12 months, with stable levodopa dosing.

## 1. Introduction

Parkinson’s disease (PD) is a prevalent neurodegenerative disorder, characterized by the progressive degeneration of dopaminergic neurons in the substantia nigra pars compacta, resulting in the classical motor triad of bradykinesia combined with either tremor, rigidity, or both. PD extends far beyond motor manifestations, encompassing a complex spectrum of non-motor symptoms (NMS) including cognitive impairment, sleep disorders, autonomic dysfunction, and mood disturbances that significantly impact patients’ quality of life (QoL) and functional capacity.

Levodopa/dopa decarboxylase inhibitor (DDCi) remains the gold standard for symptomatic treatment of PD. However, as the disease progresses, the therapeutic window for levodopa narrows, and patients develop motor complications, including wearing-off and dyskinesias that can emerge within 2 years of treatment in 50% of patients [1,2] or even earlier after 9 months of high levodopa doses [3].

Critical evidence established a clear relationship between high levels of levodopa exposure and risk of motor complications. The STRIDE-PD study demonstrated that higher levodopa dose (>400 mg/day) was among one of the predictive factors of developing dyskinesia and wearing-off [4]. Also, motor fluctuations and dyskinesia have been associated with longer disease duration and higher levodopa daily dose rather than with the duration of levodopa/DDCi therapy [5]. These findings justified the practice of using the lowest possible levodopa doses instead of withholding levodopa/DDCi therapy to provide optimal symptom control, an approach involving the early introduction of adjuvant therapies that can enhance dopaminergic efficacy.

Catechol-O-methyltransferase (COMT) inhibitors are a cornerstone of this rational polytherapy approach by blocking the peripheral metabolism of levodopa, thereby extending its half-life and providing more stable plasma concentrations available to cross the blood brain barrier (BBB) and reach the brain, where its efficacy can be further optimized by other available therapeutic options, if needed. Among the available COMT inhibitors, significant differences exist in their clinical profiles. Entacapone, despite its established efficacy, requires multiple daily doses and provides modest improvements in daily OFF time [6,7]. Tolcapone, while more potent, carries the burden of potential hepatotoxicity requiring regular monitoring [8]. Opicapone, the third-generation COMT inhibitor, offers distinct advantages including once-daily dosing, sustained enzyme inhibition, and an improved safety profile without hepatotoxic concerns [9,10]. Clinically, the ADOPTION study program demonstrated that introducing opicapone was more effective at reducing OFF time than increasing 100 mg levodopa dose in PD patients with early signs of wearing-off [11,12].

Evidence is growing about the benefit of earlier versus delayed initiation of COMT inhibitors in levodopa-treated patients with fluctuations [11,12,13,14,15,16,17]. In the case of opicapone, available data suggest a potential benefit of initiating treatment within 2 years of motor fluctuations onset [14]. The optimization of dopaminergic therapy demonstrated in several studies in PD with motor fluctuations [17,18,19,20] suggests a class effect that extends beyond motor symptom control, something that has been progressively confirmed by recent studies with opicapone [21].

The REONPARK study is a prospective, multicenter, observational study designed to evaluate the real-world effectiveness and safety of COMT inhibitors in patients presenting with early motor fluctuations. The initial 3-month interim analysis demonstrated significant improvements in motor symptoms and complications, OFF time reduction, and the quality of ON time, with minimal changes in levodopa dosing requirements [17], supporting benefits in initial motor fluctuations. The present manuscript reports updated baseline analyses and, for the first time, presents 12-month data, which extends the observation period to provide long-term data on the sustained effectiveness of COMT inhibition in this early fluctuating population.

## 2. Materials and Methods

### 2.1. Study Design

The methods of the REONPARK study have been published [17] and are discussed herein. The registry started in January 2022, and site selection was expected to remain open for 3 years. Approximately 10 sites per year were expected to participate and provide 10 patients each during the 1-year recruitment period. Recruitment was opened for new sites to join the study until July 2024, with a target of approximately 267 patients in 26 different sites.

The follow-up period was 2 years, including 5 routine visits at 3 (±1), 6 (±2), 12 (±2), 18 (±2), and 24 (±2) months after initiation with COMT inhibitor therapy (tolcapone, entacapone, or opicapone). The study was designed to reflect clinical practice as closely as possible, so there was no predefined allocation ratio for the three COMT inhibitors. All treatment decisions were made at the discretion of the participating neurologist according to clinical practice and individual patient characteristics. This pre-planned second interim analysis (database lock 15 October 2024) included all patients who had completed at least the month 3 visit, with additional data available for those who reached month 6 and month 12 of follow-up. Because recruitment and follow-up were ongoing, patients had different durations of follow-up at the interim database cut-off, and not all patients had yet reached the 12-month visit.

The study protocol was approved by the independent ethics committee of the Hospital Universitario La Princesa (Madrid, Spain). This study was conducted following the ethical principles outlined in the Declaration of Helsinki, and patients or their legal representatives provided their written informed consent to participate in the study. The study was registered in the Spanish Registry of Clinical Studies (REec, https://reec.aemps.es/reec/public/web.html; 18 February 2022) under the registration number 0012-2022-OBS.

### 2.2. Patients

Eligible patients were adults diagnosed with idiopathic PD according to the UK PD Society Brain Bank (2006) or MDS clinical diagnostic criteria (2015), treated with levodopa/dopa decarboxylase inhibitors (DDCIs), with signs of end-of-dose motor fluctuations for <2 years, and scores ≥2 for the 19-Symptom Wearing-off Questionnaire (WOQ-19). Patients with any form of Parkinsonism other than idiopathic, who met the criteria for dementia as per the investigator’s judgment, and participants involved in a clinical trial at the time of study enrollment were excluded. There was no separate formal screening phase. Patients were considered for enrolment only after the treating neurologist had made the clinical decision to initiate a COMT inhibitor, independently of study participation. Patients who met the eligibility criteria and provided written informed consent were enrolled; therefore, no screening-failure population was defined or recorded.

### 2.3. Assessments and Outcomes

Treatment response was evaluated at 3, 6, and 12 months through both Clinician Global Impression of Change (CGI-C) and Patient Global Impression of Change (PGI-C) scales. The primary disease burden assessment utilized the Movement Disorder Society-sponsored revision of the Unified Parkinson’s Disease Rating Scale (MDS-UPDRS) [22], a comprehensive 4-part rating instrument covering non-motor experiences of daily living (Part I), motor experiences of daily living (Part II), motor examination (Part III), and motor complications (Part IV). Mean daily ON time and OFF time were calculated based on question 4.3 of Part IV. During the physician-administered assessment, investigators recorded the patient-reported estimate of the number of hours spent in the OFF state and the total hours awake. Quality of life was measured using the Parkinson’s Disease Questionnaire-8 (PDQ-8), ranging from 0 to 100, with higher scores indicating worse quality of life [23]. Non-motor symptoms were assessed using the Non-Motor Symptoms Scale (NMSS), evaluating the severity and frequency of 30 non-motor symptoms across nine distinct domains, including cardiovascular, sleep/fatigue, mood/cognition, perceptual problems, attention/memory, gastrointestinal, urinary, sexual function, and miscellaneous symptoms [24]. The presence and characteristics of wearing-off phenomena were captured through WOQ-19, comprising a checklist of 19 symptoms (9 motor and 10 non-motor) that patients identified as present and assessed for improvement with subsequent anti-Parkinson medication doses [25]. The decision to deploy each scale was done according to the usual clinical practice of each site. Safety of COMT inhibitors was assessed based on the occurrence of adverse events (AEs) in the 12 months of follow-up.

### 2.4. Statistical Analysis

For the overall study, the sample size was determined using a precision-based approach for the primary descriptive objective. A total of 267 evaluable patients was estimated to provide a precision of ±6 percentage points at a two-sided 95% confidence level. Analysis populations were defined separately for each effectiveness outcome according to data availability and could therefore differ from the number of patients completing each study visit. Descriptive analyses included all patients with an available assessment at the corresponding visit, whereas repeated-measures analyses included patients with complete data for the corresponding outcome across the analyzed visits.

Descriptive statistics of quantitative data are presented as mean ± standard deviation (SD) and 95% confidence intervals (CI) and median with interquartile range (IQR), while qualitative variables were expressed as absolute and relative frequencies.

Baseline levodopa total daily dose and levodopa equivalent daily dose (LEDD) calculations incorporated all anti-PD treatments and dosing frequencies at inclusion, with post-treatment changes presented as mean absolute and relative changes using the conversion formula proposed by Jost et al. [26].

Scale-based effectiveness outcomes for MDS-UPDRS, NMSS, PDQ-8, and WOQ-19 were analyzed at months 3, 6, and 12. In addition, frequency distributions were analyzed for patients experiencing OFF periods, fluctuation-related functional impact, and dyskinesia-related functional impairment, as assessed by MDS-UPDRS Part IV.

Differences in the mean OFF time between baseline and month 12 were analyzed by paired Student’s *t*-test among patients with evaluable OFFtime data at both assessments, and the impact of motor fluctuations was also analyzed using the Stuart–Maxwell test. Repeated-measures ANOVA in patients who completed the 12 months of follow-up was performed to study the evolution of the rating scale scores over 12 months, followed by Tukey’s HSD post hoc tests. For NMSS, a subgroup analysis was performed in patients with a total score at baseline >30 (moderate burden) and >40 (severe burden), following the categories established by Ray Chaudhuri et al.: 0 (no NMS), 1–20 (mild), 21–40 (moderate), 41–70 (severe), and ≥71 (very severe) [27]. Since COMT inhibitors extend levodopa’s half-life and reduce peak–trough fluctuations [10], a logistic regression model was performed to study the effect of levodopa doses (categorized as <4.5 mg/kg/day (reference); 4.5–7.5 mg/kg/day; and >7.5 mg/kg/day to compare lower, intermediate and higher exposure groups). This analysis was performed in the subset of patients who had reached the 12-month visit. Patients with missing body weight data were excluded from the denominator of this weight-adjusted analysis. These cut-offs are not validated clinical thresholds and were used only to facilitate exploratory interpretation of weight-adjusted levodopa exposure. Logistic regression was used to examine the association between these categories and MDS-UPDRS Part IV improvement at month 12. The model was unadjusted and included levodopa dose category as the only predictor. MDS-UPDRS Part IV improvement was defined as a reduction ≥0.9 points, corresponding to the published MCID [28]. The analysis was hypothesis-generating and was not intended to establish a dose–response relationship or treatment thresholds.

Analyses of individual NMSS domains, PDQ-8 items, WOQ-19 symptoms, and subgroups defined according to baseline NMSS or levodopa dose were exploratory and hypothesis-generating. No adjustment for multiple comparisons was applied to these analyses. Safety analyses were descriptive.

No imputation of missing data was performed. Descriptive analyses were based on all available observations for each variable and visit. For paired longitudinal comparisons, only participants with non-missing data at both time points included in the relevant comparison were analyzed; therefore, the number of evaluable participants could differ across outcomes and visits. Statistical significance was set at *p*-value < 0.05. All analyses were performed using R version 4.2.0. statistical software.

## 3. Results

### 3.1. Patient Baseline Characteristics

**Figure 1:**
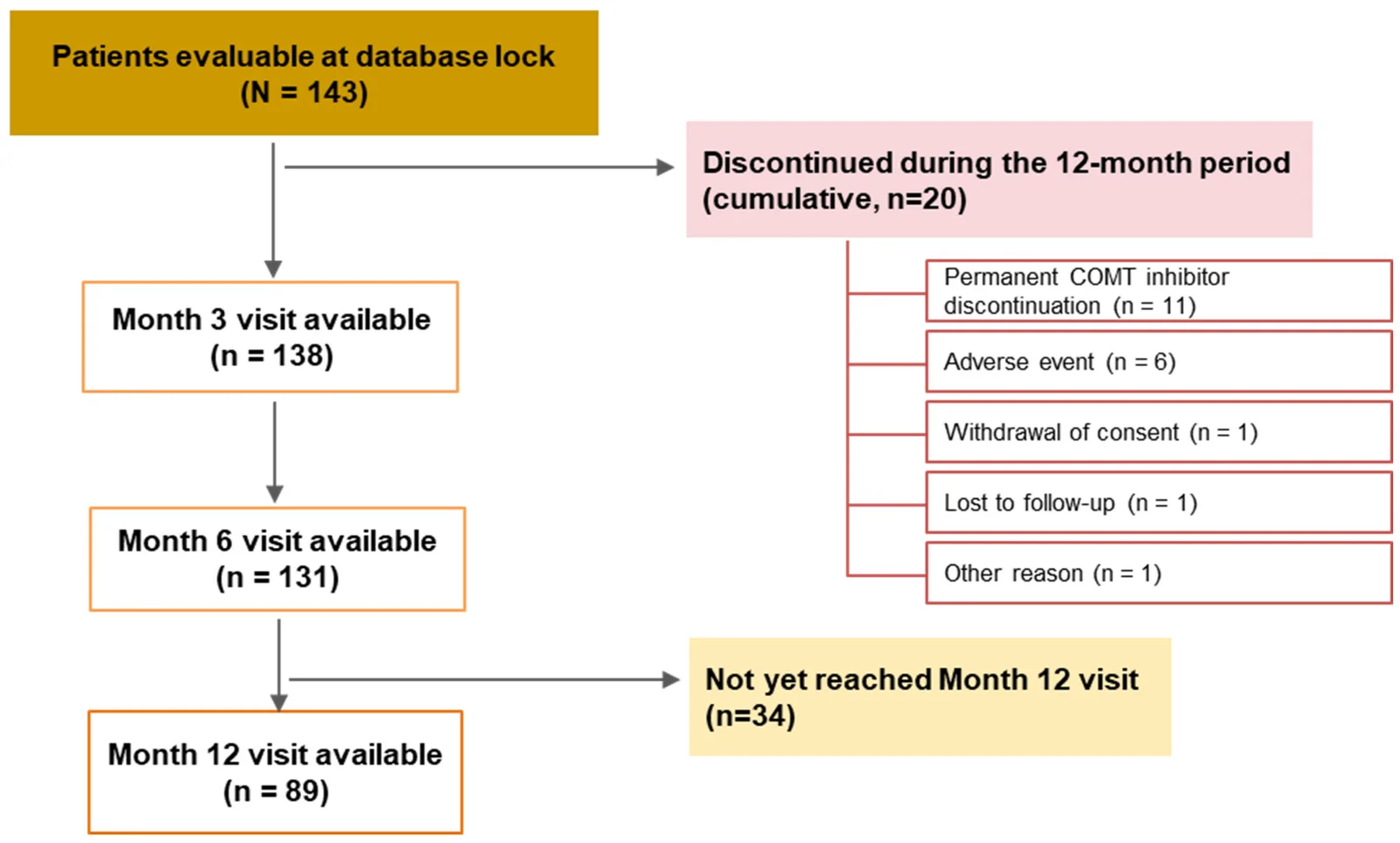
Patient flow throughout the REONPARK study. The numbers at each visit represent patients with available assessments at the corresponding time point. Because recruitment and follow-up were ongoing at the interim database lock, not all enrolled patients had reached the later study visits at the time of this analysis.

A total of 143 patients were evaluable at data cut-off. Of these, 138, 131, and 89 patients had completed the 3-, 6-, and 12-month follow-up, respectively, while 20 had discontinued the study during the first 12 months (Figure 1). The mean age at inclusion was 65.4 ± 9.9 years (65.7% male). The mean time since PD diagnosis was 5.2 ± 3.5 years, and the time since the onset of motor fluctuations was 0.6 ± 0.5 years. Disease severity at inclusion was rated by clinicians as mild-to-moderate in 108 patients (76.5%).

Patients had been receiving levodopa for a mean of 44.8 ± 28.3 months (3.7 years). The mean daily levodopa dose at inclusion was 467.1 ± 198.6 mg, mainly distributed in three (62.7% of patients) and four intakes (26.1% of patients), and the mean LEDD was 864.9 ± 332.6 (Table 1). A total of 113 patients (79%) and 80 (56%) were taking MAO inhibitors and dopamine agonists (DA), respectively, as another anti-PD medication. At inclusion, 142 patients (99.3%) initiated COMT inhibition with opicapone and one patient with a triple combination containing levodopa/carbidopa/entacapone (LCE).

### 3.2. Changes in Levodopa Dosing and Other Anti-PD Medication

After 3, 6, and 12 months of opicapone, levodopa dose remained stable in 107/138 (77.5%), 105/126 (83.3%), and 65/89 (73.0%) patients, respectively. The mean daily levodopa dose was 487.3 ± 205.6 at month 3, 495.0 ± 216.9 at month 6, and 513.5 ± 220.3 at month 12, with a mean percentage of change in total daily dose between visits of 6.6%, 2.8%, and 6.7%, respectively. Mean LEDD (including opicapone) was 890.6 ± 341.7 mg at month 3, 892.1 ± 350.2 mg at month 6, and 934.8 ± 354.0 mg at month 12, with a percentage of change in LEDD from baseline of 3.2%, 3.8%, and 10.2%, respectively.

At month 3, 111 patients (80.4%) were taking MAO inhibitors and 80 (58%) DA as another adjunct levodopa medication. These proportions remained stable at months 6 and 12: MAO inhibitors were prescribed in 106 (80.9%) and 71 (79.8%) patients, respectively, while DA was used in 73 (55.7%) and 47 (52.8%). After 7 months, the patient taking LCE switched to opicapone due to an AE (orthostatic hypotension).

### 3.3. Effectiveness

#### 3.3.1. Rating Scale Outcomes

**Figure 2:**
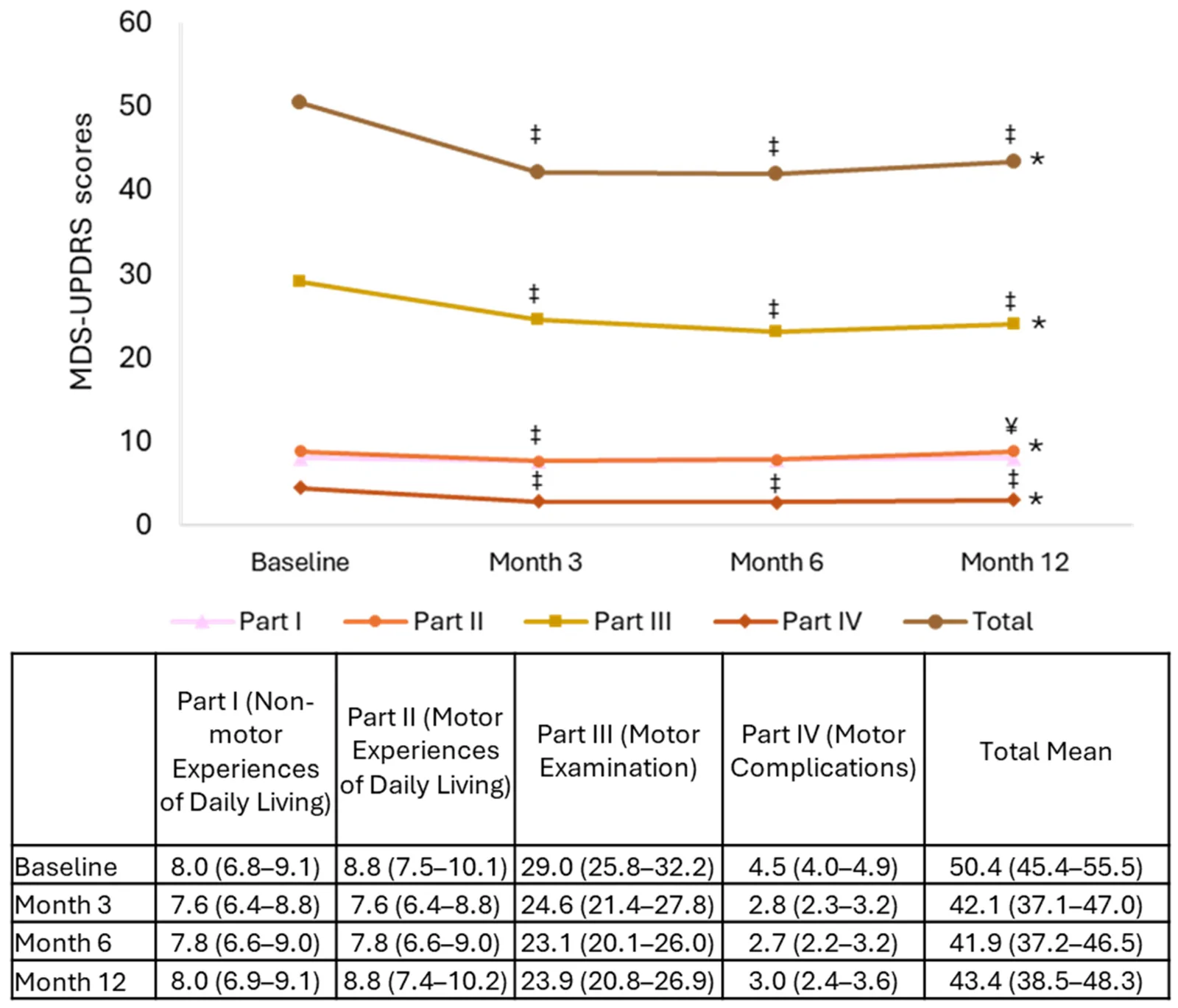
MDS-UPDRS change over 12 months. Data are expressed as mean (95% CI). * Statistically significant change over 12 months by ANOVA in patients who completed the 12 months of follow-up. ‡ Statistically significant changes from baseline by Tukey’s HSD post hoc tests. ¥ Statistically significant changes between month 3 and month 12 by Tukey’s HSD post hoc tests. CI, confidence interval; MDS-UPDRS, Movement Disorder Society–Unified Parkinson’s Disease Rating.

Table S1 shows the mean MDS-UPDRS scores from Parts I, II, III, and IV and the total score at baseline and follow-up visits. ANOVAs including only patients with complete 12-month follow-up showed statistically significant changes over time in MDS-UPDRS Part II, Part III, Part IV, and total scores (Figure 2). Post hoc comparisons revealed significantly lower scores for Part II at month 3 from baseline (mean reduction of 1.2; *p* = 0.034); however, this improvement was not maintained at month 12. In contrast, Part III, Part IV, and total score were significantly lower at months 3, 6, and 12 compared with baseline (*p* < 0.001 for all comparisons). The absolute change from baseline at 3, 6, and 12 months in Part III was 4.4, 5.9, and 5.1 points, respectively, and in Part IV was 1.7, 1.8, and 1.5 points, respectively, but no significant differences were observed between follow-up visits. Mean baseline scores of 8.0 for MDS-UPDRS Part I remained stable (mild severity) throughout the 12 months of follow-up.

In the post hoc exploratory analyses by levodopa dose, patients receiving <4.5 mg/kg/day (*n* = 24) (mean daily levodopa dose 305 mg) had higher odds of MDS-UPDRS IV improvement at 12 months after COMT inhibitor initiation compared with those receiving 4.5–7.5 mg/kg/day (*n* = 33) (OR 0.27; 95% CI 0.07–0.91; *p* = 0.043) and >7.5 mg/kg/day (*n* = 14) (OR 0.19; 95% CI 0.04–0.86; *p* = 0.037).

**Figure 3:**
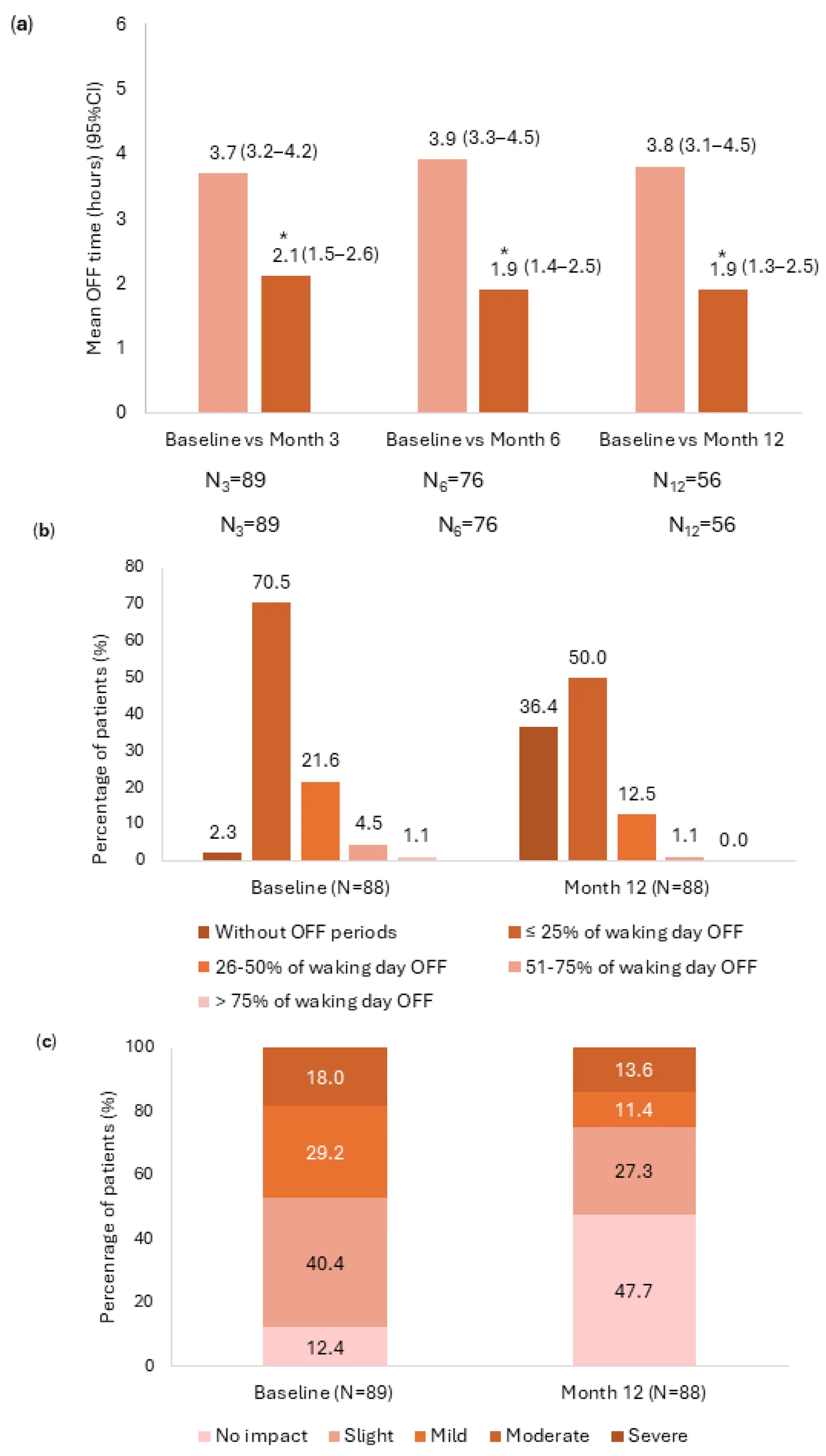
(**a**) The mean OFF time (hours) at baseline, 3, 6, and 12 months represents the investigator-recorded estimate of the number of hours spent in the OFF state during awake hours. The bars represent the mean (95% CI). *p* values derived from paired Student’s *t*-test comparisons vs. baseline and included only patients with evaluable OFF time data at both baseline and the corresponding follow-up visit: (*n* = 89 at month 3, *n* = 76 at month 6, and *n* = 56 at month 12). * *p* < 0.001. (**b**) The distribution of time spent in OFF state at baseline and month 12 among patients with available data for the corresponding MDS-UPDRS Part IV item. The values are shown as percentages. (**c**) The distribution of the functional impact of motor fluctuations at baseline and month 12 among patients with available data for the corresponding MDS-UPDRS Part IV item. The values are shown as percentages. Overall comparison was performed using the Stuart–Maxwell test. Because the analyses were based on different MDS-UPDRS Part IV variables, their sample sizes may differ. No patients reported severe impact at baseline and 12 months. CI, confidence interval.

At baseline, 99.3% of patients reported ON time with no or minimal functional impact from dyskinesia. At months 3, 6, and 12, these proportions were 99.2%, 98.4%, and 97.7%, respectively. Mean OFF time was significantly reduced from baseline to months 3, 6, and 12. Reductions ranged from 1.6 to 2 h (Figure 3a). The percentage of patients without OFF periods was 26.3% at 3 months, 28.5% at 6 months, and 36.4% at 12 months (Figure 3b). The percentage of patients reporting no impact of motor fluctuations increased from baseline (12.4%) to the 12-month follow-up visit (47.7%) (Figure 3c). At 3 months, nearly half of the patients reported no impact (47.8%), similar to that reported at 6 months (46.8%).

Mean NMSS scores remained relatively stable across visits when considering all available patients at each visit (34.6 ± 33.5 at baseline vs. 30.1 ± 31.3 at month 12) (Table 2). Repeated-measures ANOVA including only patients with complete 12-month follow-up showed a statistically significant increase over time (26.2 ± 21.9 at baseline vs. 33.1 ± 29.6 at month 12; *p* < 0.05). Post hoc analysis revealed significant differences only between month 3 and month 12 (*p* = 0.042). In exploratory analyses of patients with higher baseline non-motor symptom burden, nominal reductions were observed in selected NMSS domains. Among patients with baseline NMSS scores >30 (*n* = 27), the sleep/fatigue domain decreased from a median (IQR) of 7 (4–12) at baseline to 4 (1–9) at month 12 (*p* < 0.005), and the sexual function domain decreased from 4 (1.5–10.5) to 2 (0–5) (*p* < 0.05). Among patients with baseline NMSS scores >40 (*n* = 15), the sleep/fatigue domain decreased from 12 (5.5–16) to 4 (1.5–13) (*p* < 0.05), and the urinary domain decreased from 9 (3.5–11.5) to 3 (0–7) (*p* < 0.05). These subgroup analyses were not adjusted for multiple comparisons. Additional exploratory analyses showed changes in the cardiovascular and miscellaneous domains of the NMSS. The cardiovascular domain showed a mean (95% CI) percentage change from baseline to month 3 of 38.9 (13.7–64.2; *p* = 0.014), while the miscellaneous domain demonstrated a mean (95% CI) percentage change from baseline to month 6 of 49.5 (8.0–90.9; *p* = 0.020).

Total PDQ-8 scores were 6.6 ± 5.2 at baseline and 5.1 ± 4.8 at 12 months (Table 2). Repeated-measures ANOVA including patients with complete follow-up did not reach statistically significant changes over the 12-month period in the total scores (5.8 ± 4.9 at baseline vs. 5.1 ± 5.0 at month 12; *p* = 0.154). In an exploratory item-level analysis that was not adjusted for multiple comparisons, a nominal reduction was observed in the “felt depressed” item (0.9 ± 1.1 at baseline vs. 0.7 ± 1.0 at month 12; *p* < 0.001), with nominally lower scores at months 6 and 12 compared with baseline.

#### 3.3.2. Presence of Wearing-Off Symptoms

**Figure 4:**
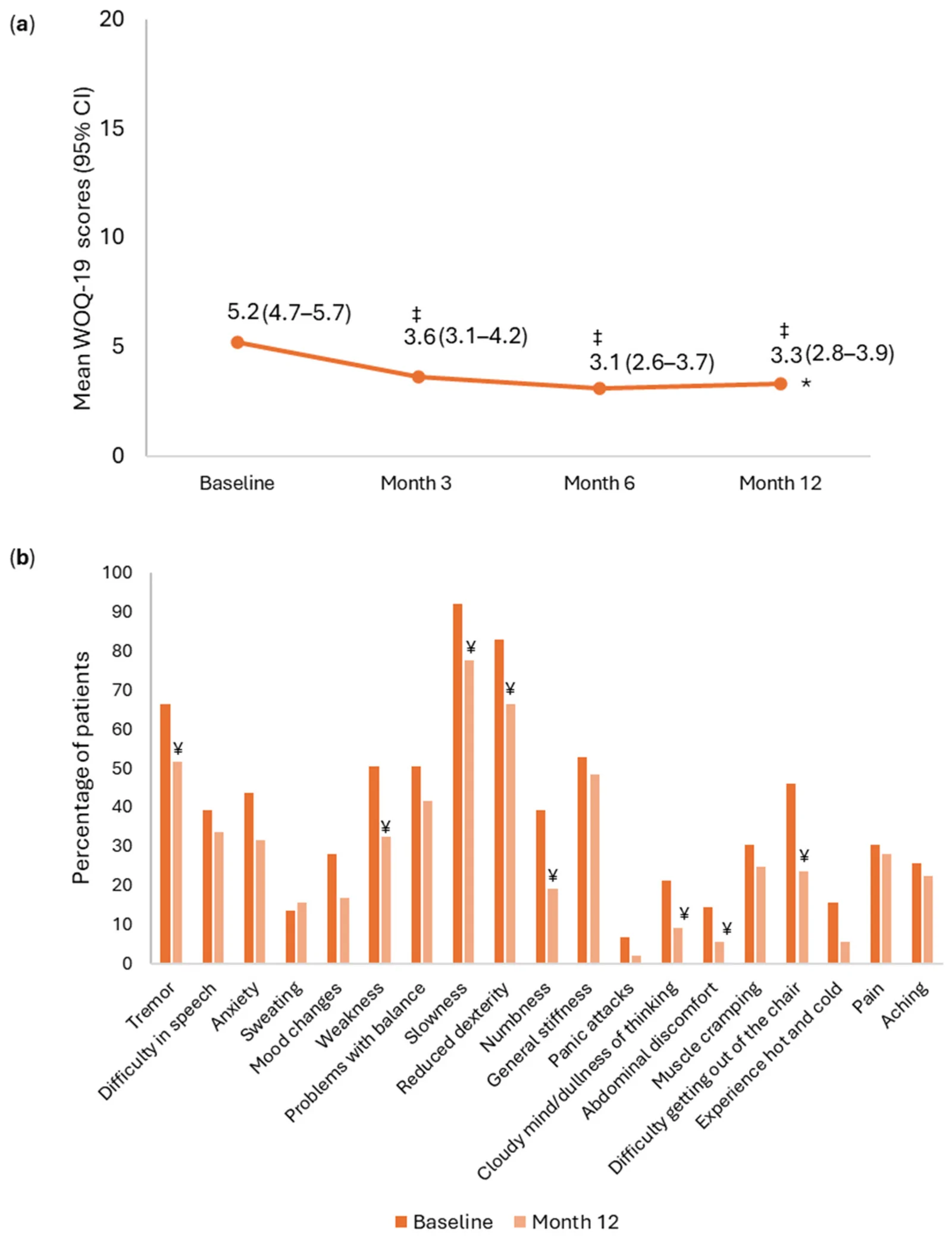
(**a**) WOQ-19 changes over 12 months. (**b**) The presence of wearing-off symptoms at baseline and 12 months. * Statistically significant change over 12 months by ANOVA in patients who completed the 12 months of follow-up (N = 82). ‡ Statistically significant changes from baseline by Tukey’s HSD post hoc. ¥ Nominal changes in the percentage of patients experiencing individual WO symptoms according to McNemar tests; these symptom-level comparisons were exploratory and were not adjusted for multiplicity. CI, confidence interval; WO, wearing-off; WOQ-19, 19-Symptom Wearing-off Questionnaire.

WOQ-19 scores reported by patients were 5.1 ± 2.2 at baseline and 3.3 ± 2.6 at 12 months. ANOVA including only patients with completed 12-month follow-up showed a statistically significant change over 12 months in WOQ-19 scores (from 5.2 ± 2.3 at baseline to 3.3 ± 2.6 at month 12; *p* < 0.001) (Figure 4a). Post hoc comparisons revealed significantly lower scores also at months 3 and 6 compared with baseline (*p* < 0.001 for all), with no significant differences between follow-up visits. The proportions of patients reporting the presence of symptoms on WOQ-19 are shown in Figure 4b. In exploratory symptom-level analyses, tremor, weakness, slowness, reduced dexterity, numbness, cloudy mind/dullness of thinking, abdominal discomfort, and difficulty getting out of the chair were the symptoms that showed nominal reductions from baseline to month 12 (Table S3).

#### 3.3.3. Clinician and Patient Global Impressions of Change

After 3 months (*n* = 137) of opicapone initiation, clinicians indicated substantial/great improvement in 39.8% of patients and slight improvement in 34.6%, with 19.5% showing no changes, minimally worse in 3.8% and much worse in 2.3%. Similar rates were observed at 6 months (*n* = 131) (29% substantial/great improvement, 29% slight improvement, 32.3% no change, minimally worse in 8.9% and much worse in 0.8%) and 12 months (*n* = 88) (28.4%, 19.3%, 30.7%, 20.5% and 1.1%, respectively). The patients’ self-rated improvement was in line with the clinicians’ impression of change: after 3 months (*n* = 137) of opicapone initiation patients reported 30.8% substantial/great improvement, 32.3% slight improvement, 25.6% no change, minimally worse in 6.8% and much worse in 3.8%; after 6 months (*n* = 130), 22.9% substantial/great improvement, 29.5% slight improvement, 33.6% no change, minimally worse in 11.5% and much worse in 2.5%; and after 12 months (*n* = 89) 23.8%, 26.1%, 29.5%, 15.9% and 4.5%.

### 3.4. Safety

Overall, from 143 patients at the data cut-off, 53.8% (*n* = 77) experienced any AE during the first 12 months of COMT inhibition, and 23.1% (*n* = 33) experienced AEs related to the COMT inhibitor (Table 3). Five patients (3.5%) reported serious AEs (SAEs), none of which were related to the COMT inhibitor.

The discontinuation from the study during the first 12 months of follow-up was 14.0% (*n* = 20) (Table S2). Among the 20 patients who discontinued the study prematurely the most frequent reason was permanent COMT inhibitor discontinuation (11/20, 55%). The remaining discontinuations were due to adverse events (*n* = 6), withdrawal of consent (*n* = 1), loss to follow-up (*n* = 1), and other reasons (*n* = 1).

Out of the 11 patients who discontinued COMT inhibitor permanently, four were due to AEs (three were COMT inhibitor-related and one AE was not related), four due to patient requests, two due to initiation of second-line therapy/DBS, and one for other reasons. Of the three patients who discontinued due to COMT inhibitor-related AEs, none were considered serious, and the AEs were behavioral changes (moderate severity), diarrhea (moderate severity), and pollakiuria (mild severity).

## 4. Discussion

The present analysis extends the previously published findings from the first 70 patients assessed at 3 months [17] by providing updated results in an expanded cohort and newly reporting 6- and 12-month outcomes and longitudinal trajectories over one year.

To our knowledge, REONPARK represents the largest prospective real-world cohort reported to date of patients with PD and early motor fluctuations (mean time since fluctuation onset of 0.6 years) initiating COMT inhibition, demonstrating a favorable risk–benefit profile in this challenging-to-manage population. The study placed no restrictions on the COMT inhibitor prescribed, and this real-world study has shown that in clinical practice opicapone was the COMT inhibitor selected in almost all patients, except for one patient who initiated levodopa/carbidopa/entacapone (LCE) but switched to opicapone after 7 months for safety reasons. This planned second interim analysis of the REONPARK study confirms the persistence of clinical improvements observed at 3 months [17] and demonstrates that earlier initiation of opicapone after the onset of motor fluctuations is associated with sustained maintenance of the initial improvements for up to 12 months. COMT inhibition with opicapone not only significantly improved OFF time, motor symptoms, and complications but also reduced the presence of wearing-off symptoms.

MDS-UPDRS motor examination (Part III) and motor complications (Part IV) were associated with significant improvements at month 3, and were maintained at months 6 and 12. By contrast, motor experiences of daily living, assessed using MDS-UPDRS Part II, was associated with significant improvement only at month 3 and returned to the baseline level at month 12.

The absolute change from baseline in Part III at 3, 6, and 12 months (4.4, 5.9, and 5.1 points, respectively) was over the threshold of 3.25 points accepted as the minimal clinically important difference (MCID) [29]. Likewise, the absolute changes from baseline in Part IV at 3, 6, and 12 months were 1.7, 1.8, and 1.5 points, respectively, exceeding the MCID of 0.9 points [28]. The lack of significant differences between follow-up visits reflects that the early response to adjunct opicapone remained stable and clinically relevant over time, which is an important finding given the progressive nature of the disease.

The clinical relevance of the benefits in MDS-UPDRS scores at 12 months was supported by the significant reduction in OFF time from baseline (3.8 h) (mean reduction of 1.9 h), above the 1 h MCID [30], the high proportion of patients who experienced no OFF time (36.4%), and the increase in the number of patients reporting no impact from motor fluctuations (from 12.4% to 47.7%). Moreover, the proportion of patients with ON time associated with no or minimal functional impact of dyskinesia remained consistently high (99.3% at baseline to 97.7% at month 12). Also supporting the earlier initiation of opicapone are the long-term (over 1 year) mean reduction in OFF time of >2.5 h seen in a post hoc subgroup analysis of BIPARK studies in patients with recent onset of motor fluctuations (mean of 1.0 years) [14], a previous post hoc analysis on the early use of opicapone in PD patients [13] and the ADOPTION trial where opicapone was used to treat early wearing-off signs (absolute reduction of 62 min of OFF time) [12]. Taken together, these findings suggest a favorable quality of ON time over time after opicapone initiation and reinforce the notion that ON/OFF quality is not binary—that is, motor states in PD fluctuate on a spectrum—and that improving the ON quality is as clinically relevant as reducing OFF time and OFF severity.

Of note, these outcomes were observed while most patients maintained their levodopa doses unchanged. In our study, mean levodopa doses varied between 467 mg/day and 513.5 mg/day, which, according to the definitions used in international studies such as the STRIDE-PD [4] (≤400 mg/day, 400–600 mg/day, and ≥600 mg/day for low, medium, and high doses, respectively), can be considered as medium doses. LEDD in our cohort was between 890.6 mg and 934.8 mg. In this line, the exploratory post hoc analysis suggested an association between lower weight-adjusted levodopa exposure and improvement in MDS-UPDRS Part IV. However, because the dose categories were not based on validated clinical thresholds, these findings should be considered hypothesis-generating and should not be interpreted as evidence of a dose–response relationship.

Sustained symptomatic changes following early opicapone initiation were also reflected in the significant reduction in the mean number of wearing-off symptoms reported on the WOQ-19. Similarly to what was seen with MDS-UPDRS scores, the main change occurred as early as 3 months and remained low after 12 months of treatment. This stable and sustained effect on wearing-off symptoms was achieved without an increased ON time with troublesome dyskinesias. The pivotal BIPARK trials and their open-label extensions [31,32,33], as well as the 3- and 6-month OPTIPARK [19,34], the Italian 6-month study [35], and the 6-month OPTI-ON study [21] confirmed this positive effect of opicapone on end-of-dose motor and non-motor fluctuations, but in later-stage PD. Thus, while new studies on earlier-stage PD are yielding more data about the long-term benefit of adding opicapone early in treatment, our results indicate that early fluctuating patients who initiated opicapone showed sustained improvements over 12 months of follow-up.

Clinician- and patient-rated global impressions of change remained generally favorable throughout follow-up. While substantial/great improvement was less frequently reported at later visits, a substantial proportion of patients continued to report improvement at 12 months, with a progressive shift towards ratings of no change. However, the lower number of available assessments at month 12 should be considered when interpreting these findings.

While the improvements in motor symptoms and motor complications observed following early opicapone initiation were maintained over 12 months, no overall benefit on total non-motor symptom burden was demonstrated. Exploratory analysis showed significant improvements in selected non-motor symptoms only among patients with higher non-motor symptom burden at baseline, in line with previous findings indicating that greater NMS severity at baseline is associated with larger improvements in non-motor symptoms [36]. The reason for the increase in the 12-month population cannot be established from this non-controlled observational study and may reflect disease progression, temporal variability in non-motor symptoms, intercurrent clinical factors, or selection effects. In the small and overlapping subgroups defined by baseline NMSS scores >30 and >40, reductions were observed in the sleep/fatigue, sexual function, and urinary domains. However, these findings were derived from analyses that were not adjusted for multiple comparisons and may have been influenced by regression to the mean resulting from selection based on high baseline NMSS values. Therefore, they should not be interpreted as evidence of a treatment effect on specific non-motor symptom domains. Previous studies, including OPTIPARK [19], OPEN-PD [20], OPTI-ON [21], and OASIS [18], have reported changes in non-motor symptoms or sleep-related outcomes in other study populations. Nevertheless, given the small subgroup sizes, overlapping definitions, selection based on baseline NMSS values, and multiple domain-level comparisons in the present study, our subgroup findings should be interpreted cautiously and considered complementary to the overall NMSS analysis.

NMS burden has a significant impact on QoL [36], and improvements of NMSS are associated with improved QoL in advanced PD [37]. Unlike studies in advanced disease and high-burden NMS, in which treatment with opicapone has significantly improved the QoL [19,20], as expected by the low scores in PDQ-8, quality of life scores remained stable. No significant change in overall PDQ-8 score was demonstrated over 12 months. Although an exploratory item-level analysis showed a nominal reduction in the “felt depressed” item, this secondary finding was not adjusted for multiple comparisons and should be interpreted cautiously.

Altogether, our results indicate that earlier initiation of opicapone after the onset of motor fluctuations was associated with the maintenance of the initial improvements over time. In the early disease stages, dopaminergic drugs, including levodopa/DDCi, provide optimal and stable motor control, accounting for a satisfactory recovery of motor signs [38]. Numerous studies have supported the initial findings suggesting that early, rather than delayed, addition of the COMT inhibitor entacapone to levodopa/DDCi therapy conferred a clinical benefit over levodopa [15]. Expanding on this concept, evidence from post hoc analysis of the BIPARK trials has demonstrated enhanced efficacy and a more favorable safety profile when used earlier in the disease course [11,13,14,39]. These findings further underscore the importance of the timely recognition and treatment of wearing-off to maximize long-term outcomes. In the real-life setting, the strongest clinical predictors of discontinuing opicapone therapy because of any AEs are advanced disease stage based on the Hoehn and Yahr scale score and longer duration of motor fluctuations [40]. Younger patients also seem to be more likely to maintain prolonged use of opicapone [41]. Other studies have identified age at PD onset, disease duration, motor symptom severity, and use of levodopa/DDCi with concomitant dopamine agonists as predictive factors of the safety and tolerability of opicapone [39,42].

In this report, the incidence of related AEs was consistent with the 3-month analysis and the 6-month OPEN-PD (21.2%) but lower compared with that reported in BIPARK trials (54–72%) and other previous real-life studies (about 45%) [19,40]. These differences should be interpreted cautiously, as AEs may be under-ascertained in observational studies compared with randomized clinical trials. In addition, the lower AE frequency observed in our cohort may also be related to the less advanced disease stage of our population and the low daily levodopa dose at baseline, below 500 mg, given evidence supporting improved tolerability of opicapone when introduced earlier [39]. The same consideration could explain the incidence of dyskinesias (7.0%) and other dopaminergic-related AEs, such as hallucinations (2.1%), which tend to be lower in earlier versus later stages of disease [39].

Concerning study limitations, we must acknowledge the lack of a comparative group as the main one. Although this study was designed to analyze the use of COMT inhibitors in clinical practice and therefore placed no restrictions on the COMT inhibitor prescribed, 142 of 143 patients initiated opicapone. Consequently, the findings of this interim analysis primarily describe outcomes following opicapone initiation and should not be generalized to COMT inhibitors as a class. The near-exclusive use of opicapone also precluded comparisons among individual COMT inhibitors and does not allow conclusions regarding comparative treatment selection. Also, the absence of a concurrent comparator prevents the observed changes from being attributed specifically to opicapone. Expectation effects, observer bias, regression to the mean, natural symptom variability, changes in concomitant management, and selective follow-up cannot be excluded. Selective assessment could also bias effect estimates upward. In addition, the variable availability of assessments across outcomes and visits may have introduced attrition or selection bias, although its direction and magnitude cannot be determined. OFF time duration was estimated retrospectively during the clinical assessment rather than measured prospectively using patient diaries. Outcomes were not analyzed according to levodopa formulation, precluding assessment of whether the use of immediate-release or controlled-release preparations influenced the observed findings. Safety outcomes were reported as cumulative frequencies, and the temporal distribution of AE onset was not analyzed. We must also consider that the results from this pre-planned interim analysis at 12 months reflect only half the planned 2-year follow-up and only 60% of the planned patient population, and should therefore be interpreted with appropriate caution.

## 5. Conclusions

This planned 12-month interim analysis of REONPARK confirms the early improvements seen at 3 months after opicapone initiation in patients experiencing early motor fluctuations and maintain the sustained effects over one year. When used early in PD patients with motor fluctuations, opicapone initiation was associated with reduced OFF time, improved quality of ON-time and clinically meaningful improvements in motor examination and motor complications, while levodopa doses remained unchanged in most patients. However, no overall benefit on total non-motor symptom burden was demonstrated. The observed safety profile was consistent with established clinical experience and demonstrated good tolerability over 12 months. Final data at the end of the study will determine if additional benefits may be observed at 2 years, in support of the importance of recognizing and managing motor and wearing-off symptoms early in patients with PD.

## Figures and Tables

**Table 1:** Baseline patient characteristics (N = 143).

Characteristic	Value
Age (years), mean ± SD	65.4 ± 9.9
Sex, *n* (%)	
Male	94 (65.7)
Female	49 (34.3)
Body weight (kg), mean ± SD ^a^	77 ± 14.8
Time since PD diagnosis (years), mean ± SD	5.2 ± 3.5
Time since onset of motor fluctuations (years), mean ± SD	0.6 ± 0.5
Hoehn and Yahr stage, *n* (%)	
I	26 (18.2)
II	105 (73.4)
III	12 (8.4)
PD subtype at diagnosis, *n* (%) ^b^	
Mild motor-predominant	85 (62.5)
Diffuse-malignant	6 (4.4)
Intermediate	45 (33.1)
Clinical Global Impression Severity of illness (CGI-S), *n* (%) ^c^	
Borderline	18 (12.8)
Mild	50 (35.5)
Moderate	58 (41.1)
Marked	14 (9.9)
Severe	1 (0.7)
History of dyskinesias, *n* (%)	25 (17.5)
MDS-UPDRS Part III (motor examination) score, mean ± SD	28.4 ± 14.7
MDS-UPDRS Part IV (motor complication) score, mean ± SD ^d^	4.5 ± 1.9
Wearing-off symptoms (WOQ-19 score), mean ± SD	5.1 ± 2.2
NMSS score, mean ± SD ^e^	34.6 ± 33.5
Levodopa daily dose at inclusion, (mg) mean ± SD Median (IQR)	467.1 ± 198.6 400 (300–600)
Number of daily doses of levodopa, *n* (%) ^f^	
2	4 (2.8)
3	89 (62.7)
4	37 (26.1)
5	10 (7.0)
6	2 (1.4)
LEDD, (mg) mean ± SD Median (IQR)	864.9 ± 332.6 775 (655–1020)
Levodopa treatment at inclusion, *n* (%)	
Levodopa and carbidopa (100/25 mg)	88 (61.5)
Levodopa and carbidopa (250/25 mg)	14 (9.8)
Levodopa and benserazide (200/50 mg)	40 (28.0)
Levodopa-controlled release	14 (9.8)
Adjunct treatment at inclusion, *n* (%)	
MAOI ^¶^	113 (79)
DA ^¶^	80 (55.9)
Amantadine	7 (4.9)
Anticholinergics (trihexyphenidyl)	8 (5.6)
Main Comorbidities, *n* (%)	
Hypertension	39 (27.3)
Dyslipidemia	25 (17.5)
Depression	10 (7.0)
Anxiety	8 (5.6)
Diabetes	13 (9.1)

Missing data (^a^ *n* = 26; ^b^ *n* = 7; ^c^ *n* = 2, ^d^ *n* = 5, ^e^ *n* = 27, ^f^ *n* = 1). ^¶^ MAOI: rasagiline (*n* = 50) and safinamide (*n* = 63). DA: pramipexole, *n* = 49; ropirinole, *n* = 13; rotigotine, *n* = 18. DA, dopamine agonists; IQR, interquartile range; LEDD, levodopa equivalent daily dose; MAOI, monoamine oxidase inhibitors; MDS-UPDRS, Movement Disorder Society-sponsored revision of the Unified Parkinson’s Disease Rating Scale; NMSS, Non-Motor Symptoms Scale; PD, Parkinson’s disease; SD, standard deviation; WOQ-19, 19-Symptom Wearing-off Questionnaire.

**Table 2:** Change in the scores of NMSS and PDQ-8.

	Descriptive Statistics (Available Cases)	Longitudinal Change (Repeated-Measures Model)
**NMSS**		
Baseline	34.6 ± 33.5 (N = 115)	26.2 ± 21.9 (N = 48)
3 months	30.3 ± 27.8 (N = 105)	25.7 ± 21.1 (N = 48)
6 months	32.4 ± 33.6 (N = 88)	29.2 ± 27.8 (N = 48)
12 months	30.1 ± 31.3 (N = 74)	33.1 ± 29.6 (N = 48) *
**PDQ-8**		
Baseline	6.6 ± 5.2 (N = 136)	5.8 ± 4.9 (N = 77)
3 months	6.0 ± 4.8 (N = 134)	5.4 ± 4.4 (N = 77)
6 months	5.3 ± 5.0 (N = 119)	4.9 ± 4.9 (N = 77)
12 months	5.1 ± 4.8 (N = 88)	5.1 ± 5.0 (N = 77)

Data are expressed as mean ± standard deviation. Descriptive values include all available patients at each visit. Longitudinal analysis includes only patients with complete 12-month follow-up. * Statistically significant changes versus month 3 by Tukey’s HSD post hoc tests. NMSS, Non-Motor Symptoms Scale; PDQ-8, Parkinson’s Disease Questionnaire; SD, standard deviation.

**Table 3:** Adverse events reported in the first 12 months of COMT inhibition.

	N (%) *
Any AE	
**Patients with any AE**	77 (53.8)
Patients with any AE of mild intensity	41 (28.7)
Patients with any AE of moderate intensity	29 (20.3)
Patients with any AE of severe intensity	7 (4.9)
**Serious Adverse Events (SAEs)**	
** Patients with ≥1 SAE**	5 (3.5)
SAEs related to the COMT inhibitor	0 (0)
**AEs related to COMT inhibition**	
Patients with any related AE	33 (23.1)
Total number of related AEs	56
Related to opicapone	55
Related to LCE	1
**Most frequent related AEs**	
Dyskinesia	10 (7.0)
Constipation	4 (2.8)
Somnolence	4 (2.8)
Dizziness	3 (2.1)
Confusional state	3 (2.1)
Visual hallucinations	3 (2.1)

* Percentage calculated on the overall population (N = 143). If a patient experienced AEs of different intensities, the patient was classified according to the maximum reported intensity. AEs, adverse events; LCE, levodopa/carbidopa/entacapone; SAEs, serious adverse events.

## Data Availability

The raw data supporting the conclusions of this article will be made available by the authors upon request due to proprietary information.

## Supplementary Materials

The following supporting information can be downloaded at https://www.mdpi.com/article/10.3390/brainsci16090961/s1, Table S1. MDS-UPDRS scores by visit. Table S2. Reasons for premature study discontinuation and permanent COMT inhibitor discontinuation during the first 12 months of follow-up. Table S3. WOQ-19 symptom-level analysis. Presence of wearing-off symptoms at baseline and 12 months.

## Abbreviations

The following abbreviations are used in this manuscript:

AE	Adverse event
BBB	Blood Brain Barrier
CGI-C	Clinician Global Impression of Change
CGI-S	Clinical Global Impression Severity
CI	Confidence Interval
COMT	Catechol O-methyltransferase
DDCi	Dopa decarboxylase inhibitor
IQR	Interquartile range
LCE	Levodopa/carbidopa/entacapone
LEDD	Levodopa equivalent daily dose
MAOI	Monoamine oxidase inhibitors
MDS-UPDRS	Movement Disorder Society-sponsored revision of the Unified Parkinson’s Disease Rating Scale
NMSS	Non-Motor Symptoms Scale
PGI-C	Patient Global Impression of Change
PD	Parkinson’s disease
PDQ-8	Parkinson’s Disease Questionnaire-8
QoL	Quality of life
SAE	Serious adverse event
SD	Standard deviation
WOQ-19	Wearing-off Questionnaire-19
