# Supplementary material for: Real-World Use of Opicapone in Management of Patients with Parkinson’s Disease and Early Motor Fluctuations in Spain: 12-Month Interim Analysis of REONPARK Study
Source: Brain Sci. 2026 Sep 11;16(9):961. doi: 10.3390/brainsci16090961 (PMC13604696; doi:10.3390/brainsci16090961)
Supplement: Supplementary file 1 [file brainsci-16-00961-s001.zip › brainsci-4496799-supplementary.pdf]

brain sciences

Article

Real-World Use of Opicapone in Management of Patients with Parkinson's Disease and Early Motor Fluctuations in Spain: 12-Month Interim Analysis of REONPARK Study

Lydia López-Manzanares [1,*], María Cerdán Sánchez [2], Berta Solano Vila [3,4], Tania Delgado Ballestero [5], Rocío García-Ramos [6], Juan García Caldentey [7], Ana Rodríguez-Sanz [8], Jesús Olivares Romero [9], Itziar Gaston [10], Mar Carmona-Abellán [11], Marina Mata Álvarez-Santullano [12], Raquel Vázquez Picón [13], Marta Rodríguez-De Miguel [14], Iciar Tegel Ayuela [14], Diogo Magalhães [15], Joerg Holenz [15] and on behalf of the REONPARK Study Group †

[1] Department of Neurology, Hospital Universitario La Princesa, 28006 Madrid, Spain
[2] Department of Neurology, Hospital Universitario Santa Lucia, 30202 Cartagena, Spain
[3] Department of Neurology, Hospital Josep Trueta, 17007 Girona, Spain
[4] Department of Neurology, Hospital Santa Caterina, 17004 Girona, Spain
[5] Consorci Corporació Sanitària Parc Taulí, Hospital Universitari Parc Taulí, 08208 Sabadell, Spain
[6] Department of Neurology, Hospital Clínico San Carlos, 28040 Madrid, Spain
[7] Centro Neurológico Oms 42, 07003 Palma de Mallorca, Spain
[8] Department of Neurology, Hospital Universitario La Paz, 28046 Madrid, Spain
[9] Neurology Service, Hospital Universitario Torrecárdenas, 04009 Almería, Spain
[10] Department of Neurology, Complejo Hospitalario de Navarra, 31008 Pamplona, Spain
[11] Department of Neurology, Hospital Universitario Basurto, 48013 Bilbao, Spain
[12] Department of Neurology, Hospital Universitario Infanta Sofia, 28702 S. Sebastian de los Reyes, Spain
[13] Neurology Department, Hospital Universitario Galdakao-Usansolo, 48960 Bizkaia, Spain
[14] Laboratorios Bial, S.A., 28016 Madrid, Spain
[15] Bial—Portela CA, S.A., 4785 Coronado, Portugal
* Correspondence: lydia.lopez@salud.madrid.org
† All participating investigators of the REONPARK study are listed in the Acknowledgments section.

Supplementary Tables

Table S1. MDS-UPDRS scores by visit

MDS-UPDRS scale	Mean ± SD
**Part I** (non-motor experiences of daily living)	
Baseline (N=141)	7.9 ± 5.3
3 months (N=135)	8.0 ± 5.8
6 months (N=123)	7.8 ± 5.7
12 months (N=87)	7.7 ± 5.1
**Part II** (motor experiences of daily living)	
Baseline (N=143)	9.1 ± 6.3
3 months (N=135)	8.0 ± 6.2

 https://doi.org/10.3390/brainsci16090961

6 months (N=122)	8.0 ± 6.1
12 months (N=88)	8.3 ± 6.4
**Part III** (motor examination)	
Baseline (N=143)	28.4 ± 14.7
3 months (N=135)	23.7 ± 14.4
6 months (N=121)	22.5 ± 14.3
12 months (N=86)	23.3 ± 13.6
**Part IV** (motor complications)	
Baseline (N=138)	4.5 ± 1.9
3 months (N=130)	3.0 ± 2.1
6 months (N=123)	2.9 ± 2.1
12 months (N=87)	3.0 ± 2.7
**Total** score	
Baseline (N=136)	49.8 ± 23.1
3 months (N=129)	42.5 ± 22.5
6 months (N=118)	41.9 ± 21.5
12 months (N=87)	42.2 ± 20.5

Descriptive values include available patients at each visit. MDS-UPDRS, Movement Disorder Society-sponsored revision of the Unified Parkinson's Disease Rating Scale.

Table S2. Reasons for premature study discontinuation and permanent COMT inhibitor discontinuation during the first 12 months of follow-up

**Premature study discontinuation (N=20)**	**n**
**Permanent COMT inhibitor discontinuation**	11
**Reason:**	
Related AEs	3
Non-related AEs	1
Patient request	4
Initiation of second-line therapy/DBS	2
Other reasons	1

https://doi.org/10.3390/brainsci16090961

**Adverse Events**	6
**Lost to follow-up**	1
**Withdrawal of consent**	1
**Other reasons**	1

The study was ongoing at the time of the planned interim database lock. Because patients entered the registry at different times, not all participants had reached the 12-month visit by the cut-off date. Therefore, some patients were not available for the corresponding follow-up assessment without representing a true withdrawal from study participation. COMT, catechol-O-methyltransferase; DBS, deep brain stimulation.

Table S3. WOQ-19 symptom-level analysis. Presence of wearing-off symptoms at baseline and 12 months

**Item**	**Baseline**	**Month 12**	**p-value***
**Tremor**	59 (66.3%)	46 (51.7%)	**0.004**
**Difficulty in speech**	35 (39.3%)	30 (33.7%)	0.359
**Anxiety**	39 (43.8%)	28 (31.5%)	0.063
**Sweating**	12 (13.5%)	14 (15.7%)	0.803
**Mood changes**	25 (28.1%)	15 (16.9%)	0.055
**Weakness**	45 (50.6%)	29 (32.6%)	**0.006**
**Problems with balance**	45 (50.6%)	37 (41.6%)	0.243
**Slowness**	82 (92.1%)	69 (77.5%)	**0.016**
**Reduced dexterity**	74 (83.1%)	59 (66.3%)	**0.009**
**Numbness**	35 (39.3%)	17 (19.1%)	**0.004**
**General stiffness**	47 (52.8%)	43 (48.3%)	0.596
**Panic attacks**	6 (6.7%)	2 (2.2%)	0.221
**Cloud mind/dullness of thinking**	19 (21.3%)	8 (9.0%)	**0.029**
**Abdominal discomfort**	13 (14.6%)	5 (5.6%)	**0.043**
**Muscle cramping**	27 (30.3%)	22 (24.7%)	0.473
**Difficulty getting out of the chair**	41 (46.1%)	21 (23.6%)	**0.001**
**Experience hot and cold**	14 (15.7%)	5 (5.6%)	0.052
**Pain**	27 (30.3%)	25 (28.1%)	0.823

https://doi.org/10.3390/brainsci16090961

**Aching**	23 (25.8%)	20 (22.5%)	0.689

*McNemar test. P-values <0.05 are highlighted in bold. WOQ-19, 19-Symptom Wearing-off Questionnaire.

https://doi.org/10.3390/brainsci16090961
